# Variations in the sleep-related breathing disorder index on polysomnography between men with HIV and controls: a matched case-control study

**DOI:** 10.1186/s12879-024-09322-z

**Published:** 2024-04-30

**Authors:** Yen-Chin Chen, Chang-Chun Chen, Wen-Kuei Lin, Han Siong Toh, Nai-Ying Ko, Cheng-Yu Lin

**Affiliations:** 1 College of Medicine, National Sun Yat-sen University, Tainan, Taiwan; 2 Department of Nursing, College of Medicine, National Cheng Kung University, Tainan, Taiwan; 3 Research and Development Committee, Taiwan AIDS Nurse Association, Taipei, Taiwan; 4 Department of Electrical and Computer Engineering, University of Arizona, Tucson, United States; 5 Sleep Medicine Center, College of Medicine, National Cheng Kung University Hospital, National Cheng Kung University, Tainan, Taiwan; 6 Department of Intensive Care Medicine, Chi Mei Medical Center, Tainan, Taiwan; 7 Institute of Clinical Medicine, College of Medicine, National Cheng Kung University, Tainan, Taiwan; 8 Department of Health and Nutrition, Chia Nan University of Pharmacy & Science, Tainan, Taiwan; 9 Department of Otolaryngology, College of Medicine, National Cheng Kung University Hospital, National Cheng Kung University, Tainan, Taiwan; 10 No. 70, Lianhai Road, Gushan District, 80424 Kaohsiung City, Taiwan

**Keywords:** People living with HIV (PLWH), Sleep-related breathing disorders, Central-apnea, Sleep architecture

## Abstract

**Background:**

Both sleep-related breathing disorders (SRBDs) and HIV infection can interfere with normal sleep architecture, and also cause physical and psychological distress. We aimed to understand the differences in the obstructive patterns, sleep architecture, physical and psychological distress when compared between people living with HIV (PLWH) and matched the severity of SRBDs controls.

**Methods:**

A comparative study using matched case-control design was conducted. Men with HIV infection (case group) were enrolled from 2016 to 2019. A control group with HIV seronegative men were matched for SRBDs severity, and were selected from sleep medicine center database for comparison.

**Results:**

The mean age of the 108 men (including 54 cases and 54 matched controls) was 33.75 years. Central-apnea index (CI) was higher in the case group rather than matched controls (mean CI, 0.34 vs. 0.17, *p* = 0.049). PLWH had a lower mean percentage of stage 3 sleep (10.26% vs. 13.94%, *p* = 0.034) and a higher percentage of rapid eye movement sleep (20.59% vs. 17.85%, *p* = 0.011) compared to matched controls. Nocturnal enuresis and sleepiness causing traffic accidents were more frequent complaint in PLWH compared to controls.

**Conclusions:**

Early detected SRBDs and subtypes in PLWH to begin treatment for the underlying cause could reduce the risk of sleepiness-related traffic accidents.

## Introduction

Sleep-related breathing disorders (SRBDs) is a chronic disease, it causes collapse in the upper airway while sleeping resulting in intermitted cessation or attenuation of breathing [1]. Patients with SRBDs may suffer from a spectrum of SRBDs, including obstructive sleep apnea (OSA), sleep-related hypoventilation (i.e., hypopnea or oxygen desaturation), and central sleep apnea (CSA) [1]. It has been associated with a greater risk of depression [2] and all-cause mortality risk [3]. It could cause a significant physiology and psychology impacts not only in people living with human immunodeficiency virus (PLWH) but also in general population such as abnormal sleep architecture [4], sleepiness, poor sleep quality [5], anxiety and depression [6].

CSA is less common than OSA that is characterized by observed absence airflow with a lack of inspiratory effort during sleep [7]. Those with CSA are caused by the brain failing to trigger the wake-up signal resulting in temporarily stopping breathing, which is the critical difference between OSA and CSA. Conditions that cause or lead to CSA remain unclear. Previous research indicated that patients with a certain type of neurological problems such as stroke, Parkinsonism, or substance use were highly correlated to CSA [8].

SRBDs has been observed as a highly prevalent problem among PLWH, with prevalence rates ranging between 41.0 and 44% [9–11]. In Taiwan, we surveyed 54 male living with HIV and found that 62.96% of PLWH with sleep problems were diagnosed as SRBDs [4]. A large multicenter cohort study revealed that a 1.42-fold risk of SRBDs in men living with HIV as compared to HIV-negative persons [12]. Since HIV virus is able to pass the blood-brain barrier (BBB), enter the central nervous system, and resulting in neurocognitive impairment [13]. Despite free support highly active antiretroviral therapy (HAART) in Taiwan, we found that the incidence of neurological disorders in PLWH still significantly increased from 22.16 per 1000 person-years in 2000 to 25.23 per 1000 person-years in 2010 through National Health Insurance Research Database (NHIRD) [14]. In addition, high prevalence of substance use in PLWH. A retrospective study in a sexual health clinic in London and found that subjects who self-reported substance use had higher odds of acquiring new HIV infection [15]. However, there is a lack of relevant evidence on the type of SRBDs in PLWH. In addition, there is very limited evidence for knowing what differences in a type of SRBDs between PLWH and the general population.

It has long been known that when people infected with HIV experience negative neurological complications due to HIV virus affects crucial parts of the brain resulting in poor sleep quality, anxiety and depression [16]. However, there are few studies to know the differences in the obstructive patterns, sleep architecture, physical and psychological distress when compared between PLWH and matched the severity of SRBDs controls.

## Methods

### Study design

This was a retrospective, matched 1:1 case-control study that reviewed all have done polysomnography (PSG) test HIV cases with sleep complaint admitted to an academic tertiary care center in the southern of Taiwan. Sleep complaint was defined as Chinese version Pittsburgh Sleep Quality Index (C-PSQI) scores greater than 5. This study was approved by the Ethics Committees of National Cheng Kung University Hospital (No. A-BR-109-031). This research was performed in accordance with relevant regulations. Prior to examination and data collection, the participants provided informed consent.

A total of 54 PLWH with sleep complaint were tested by PSG for further diagnosis sleep disorders from 2016 to 2018. For details about the participant recruitment process including subject sampling, enrollment, experimental protocol, and data acquisition have been described in detail previously [4]. We performed power calculations using G*Power 3.1.9.4 based on our sample size. The input parameters were set as follows: α = 0.05, sample size of 54 for both the experimental and control groups. The resulting power was calculated to be 0.84.

### Matching

A total of 1,810 data were selected from a secondary database of individuals seeking effective strategies for resolving their sleep problems at a sleep medicine center in southern Taiwan between October 14, 2016, and August 31, 2018. Participants who had been treated for sleep-related breathing disorders or whose polysomnography data were incomplete were excluded. Additionally, we excluded subjects with repeated data (*n* = 152), females (*n* = 478), and those who were under 20 years of age or had missing age information (*n* = 72). The final number of controls for matching was 1,108 subjects from the sleep center data. These controls were frequency-matched to the PLWH group based on age (in 5-year intervals) and the severity of SRBDs, which were classified as no [apnea hypopnea index (AHI) < 5/hour], mild (AHI between 5–15/hour), or moderate to severe (AHI over 15/hour) in a 1:1 case-to-control ratio.

### Measures

#### Objective measures: PSG

Somte polysomnography V1 monitor for patients (Compumedics Sleep, Abbotsville, Australia) was applied in this study. The signals records contained brain wave (C3-A2, C4-A1, O1-O2), nasal airflow in and out as you breathe via nasal cannula, eye movement through bilateral electrooculogram, chin electromyography, electrocardiogram, breathing effort and rate via rib cage and abdominal excursion, oxygen saturation by a finger pulse oximetry and bilateral leg movement.

***a. SRBD index*** Polysomnography data was collected and digitized on a computerized polysomnography system operated by experienced technician and interpreted by accredited sleep specialist (CY Lin) in a single reading laboratory following the American Academy of Sleep Medicine (AASM) criteria. Sleep-disordered breathing was identified according to polysomnography-determined AHI ≥ 5 events/h [17]. The AHI represents the average number of apneas and hypopneas that occur per hour during sleep, and it also serves a marker to evaluate of the severity of SRBDs [18]. There were three main types of SDB as manifested in sleep apnea: OSA, CSA, and mixed sleep apnea.

***b. Sleep architecture*** The sleep architecture refers to the basic structure of the sleeping pattern, it was calculated by PSG-derived measures included total sleep time (TST) and sleep latency (SL) in minutes, the percent of TST spent in stage 1, sleep 2, stage 3, stage of rapid eye movement (REM) sleep, and sleep efficiency (SE) (%). In addition, arousal index was measured. Each sleep characteristics were described as followings:

1. TST was determined as the total amount of hours asleep in bed;
2. SL was the minutes from the time the subject got sleep onset in bed;
3. The percent of TST spent in stage 1–3 and REM sleep, was determined by the percentage of time the subject was sleeping from sleep-onset during nocturnal sleep time;
4. SE defined as the percentage of time spent asleep while in bed.

#### Subjective measures: questionnaires

***a. Chief complaints of SRBDs*** Chief complaints were evaluated with a 7-item scale with dichotomous responses (yes/no) that assessed nonrestorative sleep, snoring, morning dry mouth, hypersomnia, morning headache, nocturia and sleepwalking. Nocturia was identified as “subject was complaint that the need to get up one or more times per night to urinate” [19].

***b. Physical distress***

(1)Daytime sleepiness.

The Chinese version of the Epworth Sleepiness Scale (C-ESS) evaluates the participants’ level of daytime sleepiness with 8 items scored from 0, never with the condition, to 3, almost with the condition. The sum of 8-item scores range from 0 to 24, a total score of 10 or higher suggests daytime sleepiness [20]. Cronbach’s alpha coefficient of 0.81 in the sleep-disordered breathing subjects [20]. 

(2)Snore.

The Chinese version of Snore Outcomes Survey (C-SOS) evaluates patients’ snoring frequency, severity, and sequela with 8 items Likert scale scored from 0, worst, to 100, best. The total score is the mean of 8 items score ranges from 0 to 100, a score of 55 or lower defines as having snoring [21]. Cronbach’s alpha of 0.86 in subjects with sleep-disordered breathing [21]. 

***c. Psychological distress*** The Hospital Anxiety and Depression Scale (HADS) is used to assess psychological distress such as anxiety and depression for patients [22, 23]. The scale was designed with a special focus on specific issues and is especially relevant for use with somatic medicine.

#### Clinical indicators collection

Clinical variables related to SRBD outcomes including age, BMI, neck size, educational level, work status, comorbidities (hypertension, diabetes mellitus, hyperlipidemia and hyperuricemia), and chief sleep complaints were collected. HIV-related clinical indicators were retrieved from patients’ electronic medical records including time since HIV diagnosis, HAART use, years from HAART initiation, viral load undetectable (it was defined as when patients’ plasma viral load was fewer than 20 copies/mL), the mean cluster of differentiation 4 (CD4) value at time of examination.

### Data analysis

The data were analyzed by statistic software (R software version 4.0.3 [24, 25]).. The chi-square tests and t-tests were used in the analysis of categorical measures and continuous measures, respectively. A p-value fewer than 0.05 is considered to be statistically significant.

## Results

### Demographics

The study group consisted of 108 adults, with 54 PLWH and 54 controls. Their demographic characteristics are provided in Table 1. The age and severity of AHI were not significantly differences due to matching successfully. The demographic characteristics that differed between PLWH and controls were BMI and neck size. BMI of PLWH has an average of 3.48 kg/m^2^ less than the controls (*p* = 0.002) as well as the neck size in PLWH was smaller than the controls (*p* = 0.001).

**Table 1:** Demographic of participants (*N* = 108)

Variables	Overall	Controls (*n* = 54)	HIV (*n* = 54)	*p*
	*n* (%)			
Age (years)[ Mean, SD]	33.75, 8.94	32.59, 8.91	34.90, 8.90	0.182
AHI (event/hour)				1.000
<5	40 (37.0)	20 (37.0)	20 (37.0)	
5–15	36 (33.3)	18 (33.3)	18 (33.3)	
15–30	16 (14.8)	8 (14.8)	8 (14.8)	
>30	16 (14.8)	8 (14.8)	8 (14.8)	
Mean, SD	16.49, 21.16	18.72, 25.59	14.27, 15.45	0.276
BMI kg/m^2^ (Mean, SD)	25.48, 5.84	27.22, 6.93	23.74, 3.84	**0.002**
Neck size (cm) [Mean, SD]	37.52, 3.33	38.58, 3.81	36.45, 2.33	**0.001**
Education level				0.164
Under Senior	23 (22.1)	9 (16.7)	14 (28.0)	
University and above	81 (77.9)	45 (83.3)	36 (72.0)	
Work status				0.097
No	5 (4.7)	1 (1.9)	4 (7.4)	
Part-time	72 (67.9)	35 (67.3)	37 (68.5)	
Full-time	8 (7.5)	3 (5.8)	5 (9.3)	
Others	21 (19.8)	15 (25.0)	8 (14.9)	
Comorbidities				
Hypertension	11 (10.6)	7 (14.0)	4 (7.4)	0.275
Diabetes Mellitus	1 (0.9)	1 (1.9)	0 (0.0)	0.306
Hyperlipidemia	15 (14.2)	8 (15.4)	7 (13.0)	0.721
Hyperuricemia	10 (9.4)	7 (13.5)	3 (5.6)	0.164
Time since HIV diagnosis (year)	3.53, 3.90	na	3.53, 3.90	na
HAART use	50 (92.6)	na	50 (92.6)	na
Years from HAART initiation	2.38, 3.40	na	2.38, 3.40	na
Viral load undetectable	41 (75.9)	na	41 (75.9)	na
CD4 value at time of examination (mean, SD)	560.53, 245.40	na	560.53, 245.40	na

*Note* apnea hypopnea index, AHI; body mass index, BMI; highly active antiretroviral therapy, HAART, non-available, na

Bold font indicates statistical significance

### Objective differences in sleep architecture

**Fig. 1:**
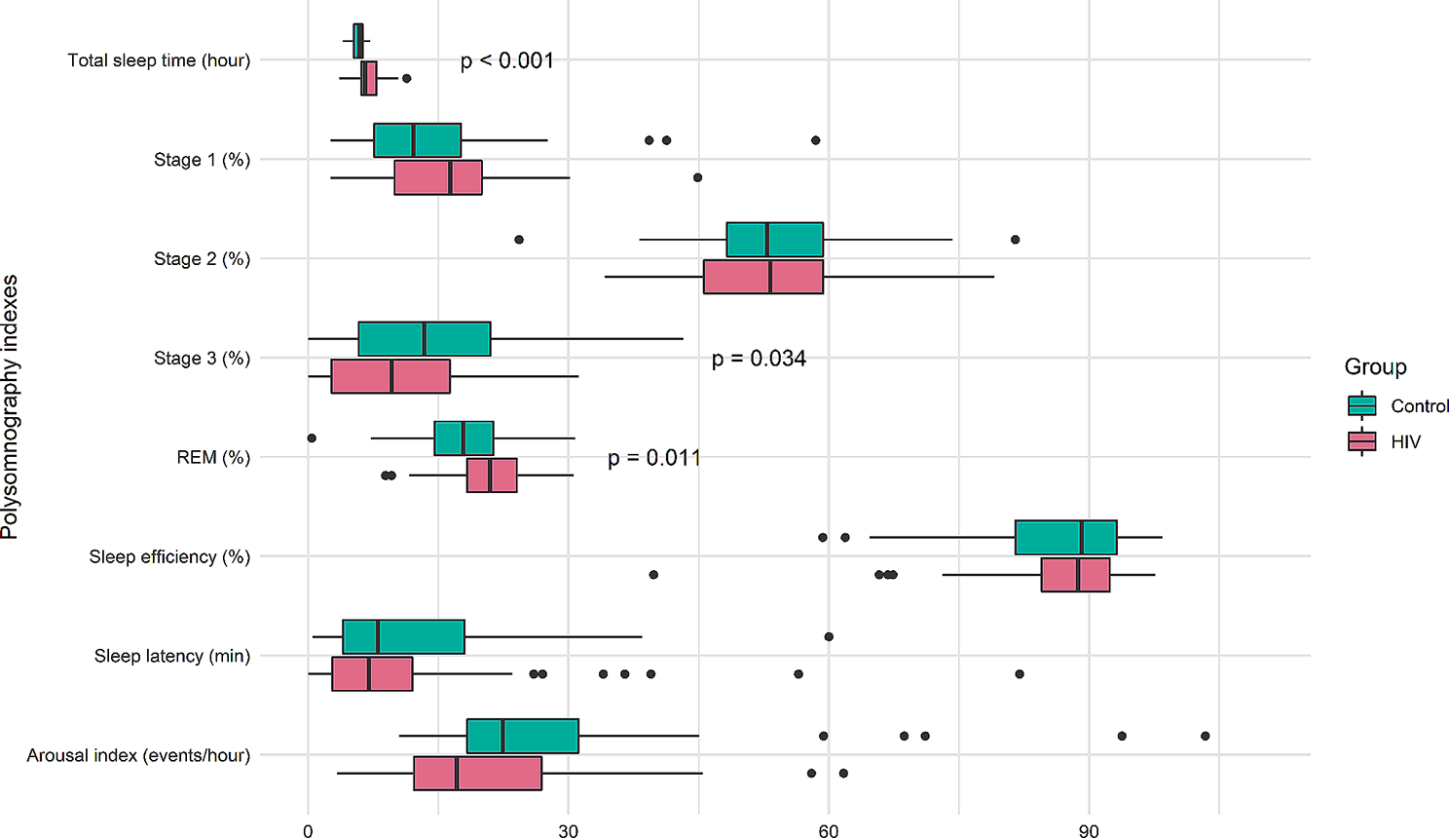
Comparison of polysomnography indexes between two group (54 HIV vs. 54 paired match group without HIV)

We examined objective differences in sleep using SRBD index (Table 2) and sleep architecture (Fig. 1). The mean central-apnea index of SRBD was higher in PLWH than the controls (0.34 vs. 0.17 events/hour, *p* = 0.049). We also saw that total sleep time, the percentage of sleep stage 3 and REM sleep, and the arousal index were significantly differences between PLWH and the controls. Total sleep time was longer in PLWH than the controls (417.05 min vs. 344.48 min, *p* < 0.001). The percentage of sleep stage 3 was lower in PLWH than the controls (10.26% vs. 13.94%, *p* = 0.034). In the opposite, the percentage of REM sleep stage was higher in PLWH than the controls (20.59% vs. 17.85%, *p* = 0.011). The mean arousal index was higher in the controls than the PLWH (28.51 vs. 20.61 events/hour, *p* = 0.012).

**Table 2:** Comparison sleep-related breathing disordered index of polysomnography between controls and HIV-infected persons

Variables	Controls (*n* = 54)	HIV (*n* = 54)	*p*
AI			0.552
Mean, SD	3.03, 9.73	4.09, 8.74	
HI			0.082
Mean, SD	15.68, 20.89	10.17, 9.80	
OI			0.421
Mean, SD	2.37, 7.24	3.61, 8.66	
CI			**0.049**
Mean, SD	0.17, 0.34	0.34, 0.52	
MI			0.413
Mean, SD	0.49, 3.07	0.14, 0.41	
ODI (events/hour)			0.541
Mean, SD	14.63, 28.02	9.15, 12.61	
Snore index (events/hour)			0.577
Mean, SD	220.48, 188.27	201.60, 161.31	

*Note* apnea index, AI; hypopnea index, HI; obstructive index, OI; central-apnea index, CI; Mixed-apnea index, MI; oxygen desaturation index, ODI

Bold font indicates statistical significance

### Differences in chief complaints of SRBDs

We compare the main sleep complaints differences between PLWH and the controls using subjective report (Table 3). We found that the top three chief complaints of SRBD no matter in PLWH or controls were snoring, non-restorative sleep, and dry mouth when waking up. PLWH is more likely to have nocturnal enuresis (*p* = 0.050) and traffic accident due to sleepiness complaints than the controls (*p* = 0.045). However, the snoring complaints is less in PLWH than the controls (*p* = 0.029).

**Table 3:** Comparison chief complaints of sleep-related breathing disorders between controls and PLWH

Variables	Controls		HIV		*p*
	*n*	%	*n*	%	
Snoring	41	78.8	32	59.3	**0.029**
Non-restorative sleep	44	84.6	40	74.1	0.181
Dry mouth when waking up	37	71.2	37	68.5	0.768
Excessive daytime sleepiness	31	59.6	27	50	0.320
Morning headache	20	38.5	14	25.9	0.167
Nocturnal enuresis	12	23.1	22	40.7	**0.050**
Sleepwalking	1	1.9	3	5.6	0.327
Traffic accident due to sleepiness	0	0	4	7.4	**0.045**

Bold font indicates statistical significance

### Subjective differences in physical and psychological distress

**Fig. 2:**
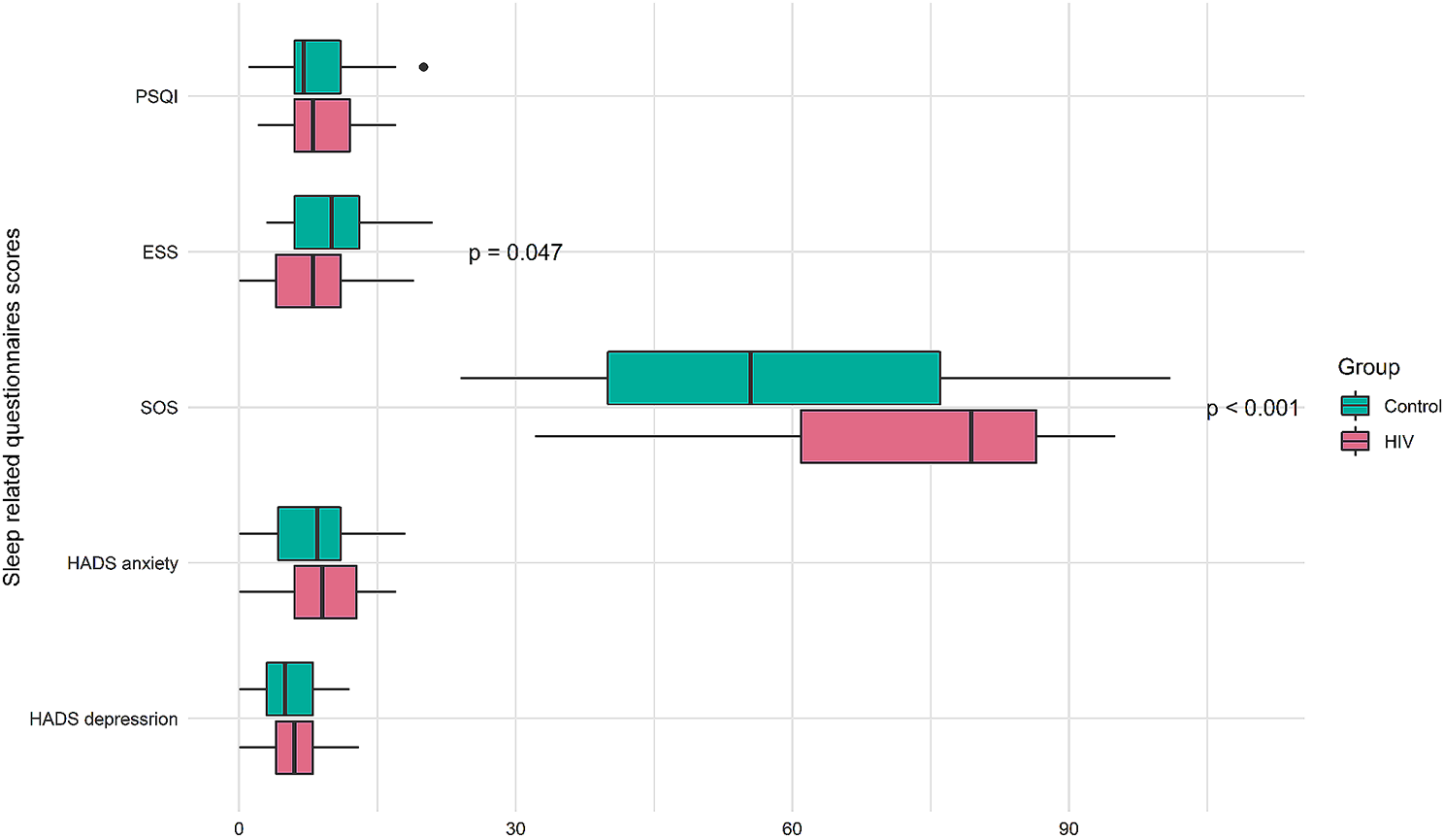
Comparison of sleep-related questionnaires scores between two group (54 HIV vs. 54 paired match group without HIV)

Our study also included several subjective measures of physical and psychological distress. There were significantly differences noted in daytime sleepiness and snore between PLWH and controls (Fig. 2). The intensity of sleepiness (mean scores 8.13 vs. 9.98, *p* = 0.047) and snore (mean scores 72.08 vs. 57.15, *p* < 0.001) were lower in PLWH than controls.

## Discussions

This is the first study to date comparing subjective and objective sleep-related data in PLWH and matched controls based on a one-night polysomnography study. We observed that PLWH had statistically significant elevations in the mean central-apnea index and the percentage of REM sleep stage, as well as reductions in the polysomnography-derived percentage of sleep stage 3 in PLWH relative to controls. When controlling study participants with a similar age and severity of SRBDs, this difference was still observed obviously. While we observed polysomnography-confirmed differences in central-apnea index between PLWH and controls, even though PLWH has achieved viral load suppression. Possible etiologies might be HIV virus does directly invade glia cells of neurons, it also triggers inflammation that may damage the brain and central nervous system [26], resulting in having more events in central-apnea and sleep-wake dysregulation [27], that would cause reductions in the percentage of sleep stage 3, more night dream, and higher arousal index. The certainty of evidence of associations of HIV infection and CSA remains unclear because of insufficient information and a limited number of polysomnography conducted in HIV population.

We identified three chief complains like nocturnal enuresis, traffic accident, and snore were significant frequency in PLWH with SRBDs compared to controls. As we know, enuresis is not just a nocturnal issue but a problem with sleep. The arousal threshold might be one of the major pathogenic factors in enuresis-nocturnal polyuria [28]. Our data also revealed the same situation. We noted that PLWH had higher mean arousal index when compared to matched controls, and tended to report higher percentage of nocturnal enuresis. In addition, we mentioned that a higher prevalence of traffic accident in PLWH than the controls. From previous literature, we have known that both SRBDs and HIV infection are associated with a significantly increased risk of traffic accidents due to repetitive hypoxia, inflammation, and sleep fragmentation resulting in sleepiness [29]. Recently, a cross-sectional study was conducted in professional drivers. The finding showed that professional drivers with HIV performed significantly poorer neurocognitive functions than those with cardiovascular diseases and healthy controls, especially in processing speed, attention and working memory [30]. It is worth to examine that the effectiveness of early detection and early treatment of SRBDs in PLWH on preventing the risk of traffic accidents.

Several previous studies reported that sleepiness, poor sleep quality, and depression were significantly increased in the PLWH as compared to the age- and sex-matched control subjects [5, 6]. However, our study had the opposite of finding and found that a statistically lower sleepiness complains in PLWH when compared to similar severity of SRBDs control. One potential explanation for the differences in sleepiness complaints between the two groups may be considering the differences in sleepiness-related parameters such as the oxygen desaturation index (ODI). In addition, the lower mean ODI in PLWH rather than in matched controls may be due to the lower BMI and neck size. This argument was similar to a previous study [29]. They compared the association between several measures of breathing patterns during nocturnal and sleepiness. The study found that nighttime oxygen desaturation severity was highly related to sleepiness [29].

Additionally, we observed that subjective difference in snoring intensity was found between PLWH and controls. The fewer PLWH quantified their intensity of snoring compared to matched similar severity of SRBDs controls. However, the data in the self-rated intensity of snoring was inconsistency with the objective snore index from PSG. It means the subjective intensity of snoring in PLWH may underreport. Similar finding was found in women [31]. A two-year observational study found the presence of snoring and self-reported intensity of snoring is underreported in women [31]. Since intensity of snoring is highly related to severity of SRBDs in HIV [4]. This difference may be one of the barriers for PLWH to reach sleep clinics and sleep laboratories for polysomnography.

Our study has several limitations. First, our results have limited generalizability to the whole HIV population and all genders because this study only covered men living with HIV. Highlight the need for future research to include a more diverse participant demographic. Second, the small sample size might be difficult to the differences related to our study outcomes. Finally, this study was limited by the selection bias of willing to have sleep study subjects that might compromise our findings.

## Conclusions

The findings from this study demonstrate that the central-hypopnea index in PLWH with SRBDs have significant difference from those of matched similar severity of SRBDs controls. We also noted that PLWH with SRBDs had lower percentage of slow sleep stage and higher percentage of REM sleep stage than matched controls. Early detected SRBDs and subtypes in PLWH to begin treatment for the underlying cause might reduce the risk of sleepiness-related traffic accidents.

## Data Availability

The datasets used and/or analysed during the current study available from the corresponding author on reasonable request.

## Declarations

### Permission to reproduce material

Control cases in this study were obtained with permission from the Sleep Medicine Center.

**Generative AI and AI-assisted technologies in the writing process**: None.

## Abbreviations

AASM: American Academy of Sleep Medicine
AHI: apnea and hypopnea index
BMI: body mass index
CD4: cluster of differentiation 4
C-ESS: The Chinese version of the Epworth Sleepiness Scale
C-SOS: The Chinese version of Snore Outcomes Survey
CSA: central sleep apnea
CPSQI: the Chinese version of the Pittsburgh sleep quality index
HAART: highly active antiretroviral therapy
HADS: Hospital Anxiety and Depression Scale
HIV: human immunodeficiency virus
ICSD-3: the International Classification of Sleep Disorders - third edition
NHIRD: National Health Insurance Research Database
OSA: obstructive sleep apnea
PLWH: people living with HIV
REM: rapid eye movement
SE: sleep efficiency
SL: sleep latency
TST: total sleep time
